# The burden of acute respiratory infections in Ecuador 2011-2015

**DOI:** 10.1371/journal.pone.0196650

**Published:** 2018-05-01

**Authors:** Wilson Chicaiza-Ayala, Aquiles R. Henríquez-Trujillo, Esteban Ortiz-Prado, Richard W. Douce, Marco Coral-Almeida

**Affiliations:** 1 OneHealth Research Group, Faculty of Health Sciences, Universidad de Las Américas, Quito, Ecuador; 2 Department of Medicine and Center for Global Health and Translational Science, State University of New York Upstate Medical University, Syracuse, New York, United States of America; 3 Department of Internal Medicine, Lakeland Health Care, St. Joseph, Michigan, United States of America; Kliniken der Stadt Köln gGmbH, GERMANY

## Abstract

**Background:**

Burden of disease studies intend to improve public health decision-making and to measure social and economic impact in population. The objective of this study was to describe the burden of acute respiratory infections (ARI) in Ecuador between 2011 and 2015.

**Methods:**

Five-year period morbidity and mortality data available from national agencies of statistics was analyzed to estimate the burden of disease attributable to acute respiratory infections. Cases and deaths registered were grouped according to their ICD-10 code into three diagnostic groups: Acute upper respiratory infections (J00-J06), Influenza and pneumonia (J09-J18), and Bronchitis and other acute lower respiratory infections (J20-J22, J85, J86). Disability-adjusted life years stratified by diagnostic and age group were calculated using the “DALY” package for R. The productivity loss in monetary terms was estimated using the human capital method.

**Results:**

Over the 5-year period studied there were a total of 14.84 million cases of acute respiratory infections, with 17 757 deaths reported (0.12%). The yearly burden of disease ranged between 98 944 to 118 651 disability-adjusted life years, with an estimated average loss of productivity of US$152.16 million (±19.6) per year. Approximately 99% of the burden can be attributed to years life lost due to premature mortality in population under 5 years old and over 60 years-old.

**Conclusions:**

The burden of acute respiratory infections remained steady during the analyzed period. Evidence-based prevention and control policies to tackle acute respiratory infections in Ecuador should focus on the population at extreme ages of life.

## Introduction

Respiratory infections are the greatest single contributor to the overall burden of disease in the world[1,2]. Despite a small decrease of 3.2%, the global burden of lower respiratory infections continues to be the third leading cause of death in 2015 worldwide[3]. According to the Global Burden of Disease study lower respiratory infections are the leading cause of Years of Life Lost (YLL) in Ecuador [3]. The Ecuadorian National Institute for Statistics and Censuses (INEC) reported Influenza and pneumonia (ICD-10 codes J09-J18) as the fifth leading cause of death[4]. Burden estimation of pneumonia and other acute respiratory infections remains an urgent need in order to evaluate their impact and to assess the cost-effectiveness of public health interventions[5].

In Ecuador, as in other tropical latitudes, the absence of a well-defined flu season, the coexistence of other respiratory viruses and the variability of clinical presentation of acute respiratory infections, predispose health services to underestimate the burden of acute respiratory infections (ARI)[6]. Despite pre-pandemic reports of viral activity in Ecuador[7] and viral identification through notification to the Ministry of Health of Ecuador, available studies do not allow the estimation of the burden of influenza or Influenza-like Illnesses (ILI) in Ecuador. Same as in ILI, definition and diagnosis of other viruses and bacterial infections is not strong enough to characterize the attribution of burden. Considering these limitations, we propose the estimation of burden of acute respiratory disease in order to assess an approximation of the impact of this set of diseases using national databases available during the 2011–2015 period.

## Materials and methods

### Geographical location and study population

Ecuador is a Latin American country located in the Pacific coast of South America. This country has a continental territory and the Galapagos Islands. The continental territory is divided by the Andes Mountains in three different regions: coastal, highlands and Amazonia. In general, there is little seasonal temperature variations through the year in each region, but there is a wide variation of climate characteristics between regions. The coast and the Amazonia are characterized as tropical rainforest and highlands as temperate regions.

INEC estimated that Ecuador population for 2016 was 16.5 million people (49.5% male and 50.5% female). Most people live in urban areas (63.3%). INEC estimated regional distribution of population as follows: Coastal 49.6%, highlands 44.7%, Amazonia 5.3% and 0.2% Galapagos Islands. 0.2% population live in non-delimited areas. Thirty percent of people are less than 15 years old. Ecuador has the highest population density in the region with an average of 54.5 habitants per km2.[8]

### Sources of information

We use available data from the national registries of deaths, hospital discharges, and ambulatory consultations published by the INEC, and the Ecuadorian Ministry of Health in the 2011–2015 period[4]. Cases and deaths registered were grouped according to their ICD-10 code (Table 1) into the following diagnostic groups: Acute upper respiratory infections (J00-J06), Influenza and pneumonia (J09-J18), Bronchitis and other acute lower respiratory infections (J20-J22)[9].

**Table 1:** Diagnoses, by ICD10 code, included in the study.

ICD-10 code	Description
**J00–J06**	**Acute upper respiratory infections**
J00	Acute nasopharyngitis (common cold)
J01	Acute sinusitis
J02	Acute pharyngitis
J03	Acute tonsillitis
J04	Acute laryngitis and tracheitis
J05	Acute obstructive laryngitis (croup) and epiglottitis
J06	Acute upper respiratory infections of multiple and unspecified sites
**J09–J18**	**Influenza and pneumonia**
J09	Influenza due to identified zoonotic or pandemic influenza virus
J10	Influenza due to identified seasonal influenza virus
J11	Influenza, virus not identified
J12	Viral pneumonia, not elsewhere classified
J13	Pneumonia due to Streptococcus pneumoniae
J14	Pneumonia due to Haemophilus influenzae
J15	Bacterial pneumonia, not elsewhere classified
J16	Pneumonia due to other infectious organisms, not elsewhere classified
J17	Pneumonia in diseases classified elsewhere
J18	Pneumonia, organism unspecified
**J20–J22**	**Other acute lower respiratory infections**
J20	Acute bronchitis
J21	Acute bronchiolitis
J22	Unspecified acute lower respiratory infection
**J85-J86**	**Suppurative and necrotic conditions of lower respiratory tract**
J85	Abscess of lung and mediastinum
J86	Pyothorax

ICD-10, International Statistical Classification of Diseases and Related Health Problems 10th Revision.

Outpatient data were obtained from the Automated Daily Register of Ambulatory Consultations (Registro Diario Automatizado de Consultas y Atenciones Ambulatorias—RDACAA) provided by the Ministry of Health since year 2013. Based on this information, an outpatient to inpatient ratio was estimated. This ratio allowed extrapolation from hospitalized cases to total cases for years 2011 and 2012[10]. Information from ambulatory consultations, hospital discharges, and deaths registries was tabulated by ICD code, sex and age group to calculate incidence and mortality rates. Incidence and mortality rates per 1000 population were calculated with a level of confidence of 95%.

Ethical approval was not required for this study. Information used in this analysis came from freely available public databases published by the INEC and the Ecuadorian Ministry of Health. Anonymity of clinical records is guaranteed by legal mandate and their use is authorized for research and academic purposes while keeping confidentiality (“REGLAMENTO DE INFORMACION CONFIDENCIAL EN SISTEMA NACIONAL DE SALUD” available at: http://instituciones.msp.gob.ec/cz6/images/lotaip/Enero2015/Acuerdo%20Ministerial%205216.pdf).

### Estimation of the burden of disease

The burden of disease attributable to acute respiratory infections for each year of the study period, was measured in disability-adjusted life years (DALYs) as summary measure following the methods described by Murray *et al*. [11]. Calculations were made using the “DALY” package for R [12].

DALYs are the sum of years lived with disability (YLDs) and years of life lost due to premature mortality (YLLs). Following an incidence perspective, YLDs were estimated as the product of the number of incident cases times the duration of disease symptoms in years, and the disability weight attributed to the disease.

To estimate the disease duration, we calculated the average hospital length of stay based on the reports obtained from the databases and from available literature [13,14]. 0.0192 years for J00-J06, 0.041 years for J20-J22 and 0.0274 years for J09-J018, J85-J86.

Disability weights (DW) for each disease group were assigned accordingly to the estimates by the Global Burden of Disease 2013 study: mild acute episode DW = 0.006 (95% CI 0.002–0.012) for ICD-10 codes J00-J06; moderate acute episode DW = 0.051 (95% CI 0.032–0.074) for ICD-10 codes J20-J22; and severe acute episode DW = 0.133 (95% CI 0.088–0.190) for ICD-10 codes J09-J18, J85-J86[15].

YLLs were estimated as the product of the number of deaths and the residual life expectancy at the age of death. To estimate residual life expectancy we used the Coale and Demeny model life table West, with a life expectancy at birth of 80 years for males and 82.5 years for females[11]. YLLs were also calculated using the WHO Life tables 2011–2015 estimates for Ecuador[16]. A time discount rate of 3% without age weighting was used in the calculations[11].

The economic burden was estimated following the human capital method[17], as the indirect costs generated by the productivity losses, valued in current US dollars, due to the absenteeism of the economically active patient, or the productivity loss of one caregiver in the case of minors and the elderly. Indirect costs included the loss of resources due to morbidity and mortality, considering the minimum monthly wage of a formal worker in Ecuador valued in US$ 516.25 [18]. It was not possible to perform a claims-based examination of health care costs because of the lack of electronic health records and billing systems in the public sector.

### Statistical analysis

The statistical differences for mortality rates between age groups were assessed using the Poisson test. The differences of the contribution of DALYs by age group were assessed using the proportion test with the “Bonferroni” correction for multiple comparisons. To assess the seasonality of events, a regression analysis using the “tslm” function of the “forecast” package in R version 3.3.3 was used. Statistical significance was assumed for all the analysis with a p-value inferior to 0.05.

## Results

Over the 5-year period studied there were a total of 14.84 million cases of acute respiratory infections with 17 757 deaths reported (0.12%). About 17 241 deaths (97.2%) were attributed to influenza or pneumonia (ICD-10 codes J09-J18). Significant differences were observed among the mortality rates across all age categories (p<0.00005). Mortality rates during the studied period were higher in the over60 years-old age group being 6 to 9 times higher than in the population under 5 years-old, the other age categories had statistically significant lower rates (p<0.00005).

Seasonality was observed for hospitalizations from August to January (p = 0.000059) and for mortality cases from September to February (p = 0.048).

There were 258 261 hospital discharges during the study period, 74.15% due to ICD-10 codes J09-J18, J85-J86. Incidence of acute upper respiratory infections (J00-J06) was higher in the age group under 5 years-old in all the periods studied. Incidence rates per 1000 population for ambulatory and hospitalized cases are presented in Tables 2 and 3 respectively, mortality rates per 1000 population are presented in Table 4.

**Table 2:** Incidence rates per thousand population of ambulatory cases of acute respiratory infections in Ecuador, years 2011–15.

Year—age group	ICD-10: J00-J06		ICD-10: J09-J18		ICD-10: J20-J22	
	cases	rate (95% CI)	cases	rate (95% CI)	cases	rate (95% CI)
**2011—total**	**3,145,286**	**206.03 (205.8 to 206.25)**	**165,876**	**10.87 (10.81 to 10.92)**	**294,525**	**19.29 (19.22 to 19.36)**
0–4 years old	1,256,336	741.28 (739.99 to 742.58)	93,761	55.32 (54.97 to 55.68)	171,122	100.97 (100.49 to 101.45)
5–14 years old	872,052	271.52 (270.95 to 272.09)	28,610	8.91 (8.81 to 9.01)	59,840	18.63 (18.48 to 18.78)
15–44 years old	709,869	101.94 (101.7 to 102.17)	22,167	3.18 (3.14 to 3.23)	32,108	4.61 (4.56 to 4.66)
45–59 years old	169,089	86.15 (85.74 to 86.56)	8,199	4.18 (4.09 to 4.27)	12,838	6.54 (6.43 to 6.65)
60 + years old	137,940	96.24 (95.73 to 96.75)	13,139	9.17 (9.01 to 9.32)	18,617	12.99 (12.8 to 13.18)
**2012—total**	**2,868,531**	**184.82 (184.6 to 185.03)**	**138,008**	**8.89 (8.84 to 8.94)**	**262,327**	**16.9 (16.84 to 16.97)**
0–4 years old	1,131,414	667.71 (666.48 to 668.95)	76,820	45.34 (45.02 to 45.66)	150,803	89 (88.55 to 89.45)
5–14 years old	793,471	244.71 (244.17 to 245.25)	23,678	7.3 (7.21 to 7.4)	53,160	16.39 (16.26 to 16.53)
15–44 years old	658,827	92.91 (92.68 to 93.13)	18,628	2.63 (2.59 to 2.66)	29,268	4.13 (4.08 to 4.17)
45–59 years old	157,463	77.86 (77.47 to 78.24)	7,053	3.49 (3.41 to 3.57)	11,788	5.83 (5.72 to 5.93)
60 + years old	127,356	86.62 (86.14 to 87.09)	11,829	8.05 (7.9 to 8.19)	17,308	11.77 (11.6 to 11.95)
**2013—total**	**2,652,401**	**168.14 (167.94 to 168.34)**	**115,632**	**7.33 (7.29 to 7.37)**	**236,257**	**14.98 (14.92 to 15.04)**
0–4 years old	1,043,257	616.8 (615.62 to 617.99)	63,596	37.6 (37.31 to 37.89)	135,658	80.2 (79.78 to 80.63)
5–14 years old	723,580	221.23 (220.72 to 221.74)	19,382	5.93 (5.84 to 6.01)	47,045	14.38 (14.25 to 14.51)
15–44 years old	620,067	85.9 (85.68 to 86.11)	15,822	2.19 (2.16 to 2.23)	26,782	3.71 (3.67 to 3.75)
45–59 years old	147,249	70.72 (70.36 to 71.08)	6,041	2.9 (2.83 to 2.98)	10,715	5.15 (5.05 to 5.24)
60 + years old	118,248	78.22 (77.78 to 78.67)	10,791	7.14 (7 to 7.27)	16,057	10.62 (10.46 to 10.79)
**2014—total**	**2,156,911**	**134.58 (134.4 to 134.76)**	**68,958**	**4.3 (4.27 to 4.33)**	**182,745**	**11.4 (11.35 to 11.45)**
0–4 years old	801,673	475.26 (474.22 to 476.3)	35,210	20.87 (20.66 to 21.09)	99,082	58.74 (58.37 to 59.11)
5–14 years old	607,707	184.4 (183.94 to 184.87)	11,961	3.63 (3.56 to 3.7)	37,783	11.46 (11.35 to 11.58)
15–44 years old	520,123	70.8 (70.61 to 71)	9,351	1.27 (1.25 to 1.3)	22,245	3.03 (2.99 to 3.07)
45–59 years old	126,308	58.97 (58.65 to 59.3)	4,068	1.9 (1.84 to 1.96)	9,308	4.35 (4.26 to 4.44)
60 + years old	101,100	64.92 (64.52 to 65.33)	8,368	5.37 (5.26 to 5.49)	14,327	9.2 (9.05 to 9.35)
**2015—total**	**2,066,451**	**126.94 (126.77 to 127.11)**	**57,921**	**3.56 (3.53 to 3.59)**	**169,318**	**10.4 (10.35 to 10.45)**
0–4 years old	789,751	469.67 (468.63 to 470.7)	29,640	17.63 (17.43 to 17.83)	94,609	56.26 (55.91 to 56.62)
5–14 years old	555,808	167.6 (167.16 to 168.04)	9,003	2.71 (2.66 to 2.77)	32,862	9.91 (9.8 to 10.02)
15–44 years old	506,947	67.84 (67.65 to 68.02)	8,065	1.08 (1.06 to 1.1)	20,500	2.74 (2.71 to 2.78)
45–59 years old	119,192	54.14 (53.83 to 54.45)	3,350	1.52 (1.47 to 1.57)	8,195	3.72 (3.64 to 3.8)
60 + years old	94,753	58.98 (58.61 to 59.36)	7,863	4.89 (4.79 to 5)	13,152	8.19 (8.05 to 8.33)

ICD-10, International Statistical Classification of Diseases and Related Health Problems 10th Revision; J00-J06, Acute upper respiratory infections; J09-J18, Influenza and pneumonia; J20-J22, Other acute lower respiratory infections.

**Table 3:** Hospitalization rates per thousand population, by age group, for acute respiratory infections in Ecuador, 2011–15.

Year—age group	ICD-10: J00-J06		ICD-10: J09-J18		ICD-10: J20-J22		ICD-10: J85-J86	
	cases	rate (95% CI)	cases	rate (95% CI)	cases	rate (95% CI)	cases	rate (95% CI)
**2011—total**	**4,900**	**0.321 (0.312 to 0.33)**	**37,178**	**2.435 (2.411 to 2.46)**	**7,441**	**0.487 (0.476 to 0.499)**	**192**	**0.013 (0.011 to 0.014)**
0–4 years old	2,425	1.431 (1.374 to 1.489)	19,445	11.473 (11.313 to 11.636)	5,490	3.239 (3.154 to 3.326)	45	0.027 (0.019 to 0.036)
5–14 years old	1,022	0.318 (0.299 to 0.338)	3,402	1.059 (1.024 to 1.095)	831	0.259 (0.241 to 0.277)	11	0.003 (0.002 to 0.006)
15–44 years old	1,022	0.147 (0.138 to 0.156)	3,636	0.522 (0.505 to 0.539)	440	0.063 (0.057 to 0.069)	55	0.008 (0.006 to 0.01)
45–59 years old	242	0.123 (0.108 to 0.14)	2,102	1.071 (1.026 to 1.118)	182	0.093 (0.08 to 0.107)	41	0.021 (0.015 to 0.028)
60 + years old	189	0.132 (0.114 to 0.152)	8,593	5.995 (5.869 to 6.123)	498	0.347 (0.318 to 0.379)	40	0.028 (0.02 to 0.038)
**2012—total**	**4,982**	**0.321 (0.312 to 0.33)**	**38,721**	**2.495 (2.47 to 2.52)**	**8,391**	**0.541 (0.529 to 0.552)**	**198**	**0.013 (0.011 to 0.015)**
0–4 years old	2,383	1.406 (1.35 to 1.464)	21,355	12.603 (12.434 to 12.773)	6,380	3.765 (3.673 to 3.859)	36	0.021 (0.015 to 0.029)
5–14 years old	1,135	0.35 (0.33 to 0.371)	3,748	1.156 (1.119 to 1.194)	989	0.305 (0.286 to 0.325)	10	0.003 (0.001 to 0.006)
15–44 years old	1,046	0.148 (0.139 to 0.157)	2,943	0.415 (0.4 to 0.43)	452	0.064 (0.058 to 0.07)	68	0.01 (0.007 to 0.012)
45–59 years old	233	0.115 (0.101 to 0.131)	1,796	0.888 (0.847 to 0.93)	159	0.079 (0.067 to 0.092)	31	0.015 (0.01 to 0.022)
60 + years old	185	0.126 (0.108 to 0.145)	8,879	6.039 (5.914 to 6.166)	411	0.28 (0.253 to 0.308)	53	0.036 (0.027 to 0.047)
**2013—total**	**6,859**	**0.435 (0.425 to 0.445)**	**42,525**	**2.696 (2.67 to 2.722)**	**8,229**	**0.522 (0.51 to 0.533)**	**166**	**0.011 (0.009 to 0.012)**
0–4 years old	3,654	2.16 (2.091 to 2.232)	22,350	13.214 (13.041 to 13.388)	6,021	3.56 (3.47 to 3.651)	24	0.014 (0.009 to 0.021)
5–14 years old	1,486	0.454 (0.432 to 0.478)	3,640	1.113 (1.077 to 1.15)	1,030	0.315 (0.296 to 0.335)	14	0.004 (0.002 to 0.007)
15–44 years old	1,215	0.168 (0.159 to 0.178)	3,735	0.517 (0.501 to 0.534)	491	0.068 (0.062 to 0.074)	66	0.009 (0.007 to 0.012)
45–59 years old	259	0.124 (0.11 to 0.14)	2,542	1.221 (1.174 to 1.269)	216	0.104 (0.09 to 0.119)	24	0.012 (0.007 to 0.017)
60 + years old	245	0.162 (0.142 to 0.184)	10,258	6.786 (6.655 to 6.918)	471	0.312 (0.284 to 0.341)	38	0.025 (0.018 to 0.035)
**2014—total**	**5,730**	**0.358 (0.348 to 0.367)**	**37,465**	**2.338 (2.314 to 2.361)**	**7,696**	**0.48 (0.47 to 0.491)**	**179**	**0.011 (0.01 to 0.013)**
0–4 years old	3,023	1.792 (1.729 to 1.857)	19,678	11.666 (11.503 to 11.83)	5,895	3.495 (3.406 to 3.585)	36	0.021 (0.015 to 0.03)
5–14 years old	1,242	0.377 (0.356 to 0.398)	3,178	0.964 (0.931 to 0.998)	714	0.217 (0.201 to 0.233)	8	0.002 (0.001 to 0.005)
15–44 years old	1,013	0.138 (0.13 to 0.147)	2,636	0.359 (0.345 to 0.373)	423	0.058 (0.052 to 0.063)	69	0.009 (0.007 to 0.012)
45–59 years old	244	0.114 (0.1 to 0.129)	1,835	0.857 (0.818 to 0.897)	189	0.088 (0.076 to 0.102)	31	0.014 (0.01 to 0.021)
60 + years old	208	0.134 (0.116 to 0.153)	10,138	6.51 (6.384 to 6.638)	475	0.305 (0.278 to 0.334)	35	0.022 (0.016 to 0.031)
**2015—total**	**5,451**	**0.335 (0.326 to 0.344)**	**34,682**	**2.13 (2.108 to 2.153)**	**7,060**	**0.434 (0.424 to 0.444)**	**216**	**0.013 (0.012 to 0.015)**
0–4 years old	3,034	1.804 (1.741 to 1.87)	17,492	10.403 (10.249 to 10.558)	5,387	3.204 (3.119 to 3.29)	28	0.017 (0.011 to 0.024)
5–14 years old	1,143	0.345 (0.325 to 0.365)	3,173	0.957 (0.924 to 0.991)	688	0.207 (0.192 to 0.224)	14	0.004 (0.002 to 0.007)
15–44 years old	890	0.119 (0.111 to 0.127)	2,310	0.309 (0.297 to 0.322)	361	0.048 (0.043 to 0.054)	86	0.012 (0.009 to 0.014)
45–59 years old	197	0.089 (0.077 to 0.103)	1,662	0.755 (0.719 to 0.792)	161	0.073 (0.062 to 0.085)	50	0.023 (0.017 to 0.03)
60 + years old	187	0.116 (0.1 to 0.134)	10,045	6.253 (6.131 to 6.376)	463	0.288 (0.263 to 0.316)	38	0.024 (0.017 to 0.032)

ICD-10, International Statistical Classification of Diseases and Related Health Problems 10th Revision; J00-J06, Acute upper respiratory infections; J09-J18, Influenza and pneumonia; J20-J22, Other acute lower respiratory infections; J85-J86, Suppurative and necrotic conditions of lower respiratory tract.

**Table 4:** Mortality rates per 10 thousand population, by age group, for acute respiratory infections in Ecuador, 2011–15.

Year—age group	ICD-10: J00-J06		ICD-10: J09-J18		ICD-10: J20-J22		ICD-10: J85-J86	
	deaths	rate (95% CI)	deaths	rate (95% CI)	deaths	rate (95% CI)	deaths	rate (95% CI)
**2011—total**	**20**	**0.013 (0.008 to 0.02)**	**3,086**	**2.021 (1.951 to 2.094)**	**80**	**0.052 (0.042 to 0.065)**	**31**	**0.02 (0.014 to 0.029)**
0–4 years old	13	0.077 (0.041 to 0.131)	369	2.177 (1.961 to 2.411)	44	0.26 (0.189 to 0.349)	-	0 (0 to 0.022)
5–14 years old	-	0 (0 to 0.011)	73	0.227 (0.178 to 0.286)	5	0.016 (0.005 to 0.036)	-	0 (0 to 0.011)
15–44 years old	2	0.003 (0 to 0.01)	251	0.36 (0.317 to 0.408)	4	0.006 (0.002 to 0.015)	7	0.01 (0.004 to 0.021)
45–59 years old	-	0 (0 to 0.019)	184	0.937 (0.807 to 1.083)	2	0.01 (0.001 to 0.037)	10	0.051 (0.024 to 0.094)
60 + years old	5	0.035 (0.011 to 0.081)	2,209	15.412 (14.776 to 16.068)	25	0.174 (0.113 to 0.257)	14	0.098 (0.053 to 0.164)
**2012—total**	**14**	**0.009 (0.005 to 0.015)**	**3,608**	**2.325 (2.249 to 2.402)**	**115**	**0.074 (0.061 to 0.089)**	**18**	**0.012 (0.007 to 0.018)**
0–4 years old	8	0.047 (0.02 to 0.093)	377	2.225 (2.006 to 2.461)	53	0.313 (0.234 to 0.409)	1	0.006 (0 to 0.033)
5–14 years old	1	0.003 (0 to 0.017)	66	0.204 (0.157 to 0.259)	5	0.015 (0.005 to 0.036)	1	0.003 (0 to 0.017)
15–44 years old	1	0.001 (0 to 0.008)	222	0.313 (0.273 to 0.357)	6	0.008 (0.003 to 0.018)	1	0.001 (0 to 0.008)
45–59 years old	-	0 (0 to 0.018)	198	0.979 (0.847 to 1.125)	6	0.03 (0.011 to 0.065)	6	0.03 (0.011 to 0.065)
60 + years old	4	0.027 (0.007 to 0.07)	2,745	18.669 (17.977 to 19.381)	45	0.306 (0.223 to 0.41)	9	0.061 (0.028 to 0.116)
**2013—total**	**20**	**0.013 (0.008 to 0.02)**	**3,754**	**2.38 (2.304 to 2.457)**	**73**	**0.046 (0.036 to 0.058)**	**22**	**0.014 (0.009 to 0.021)**
0–4 years old	9	0.053 (0.024 to 0.101)	321	1.898 (1.696 to 2.117)	26	0.154 (0.1 to 0.225)	3	0.018 (0.004 to 0.052)
5–14 years old	-	0 (0 to 0.011)	71	0.217 (0.17 to 0.274)	2	0.006 (0.001 to 0.022)	2	0.006 (0.001 to 0.022)
15–44 years old	1	0.001 (0 to 0.008)	280	0.388 (0.344 to 0.436)	3	0.004 (0.001 to 0.012)	4	0.006 (0.002 to 0.014)
45–59 years old	1	0.005 (0 to 0.027)	281	1.35 (1.196 to 1.517)	5	0.024 (0.008 to 0.056)	2	0.01 (0.001 to 0.035)
60 + years old	9	0.06 (0.027 to 0.113)	2,801	18.529 (17.849 to 19.228)	37	0.245 (0.172 to 0.337)	11	0.073 (0.036 to 0.13)
**2014—total**	**5**	**0.003 (0.001 to 0.007)**	**3,422**	**2.135 (2.064 to 2.208)**	**73**	**0.046 (0.036 to 0.057)**	**19**	**0.012 (0.007 to 0.019)**
0–4 years old	1	0.006 (0 to 0.033)	318	1.885 (1.684 to 2.104)	32	0.19 (0.13 to 0.268)	3	0.018 (0.004 to 0.052)
5–14 years old	-	0 (0 to 0.011)	47	0.143 (0.105 to 0.19)	4	0.012 (0.003 to 0.031)	3	0.009 (0.002 to 0.027)
15–44 years old	-	0 (0 to 0.005)	227	0.309 (0.27 to 0.352)	4	0.005 (0.001 to 0.014)	3	0.004 (0.001 to 0.012)
45–59 years old	-	0 (0 to 0.017)	215	1.004 (0.874 to 1.147)	6	0.028 (0.01 to 0.061)	-	0 (0 to 0.017)
60 + years old	4	0.026 (0.007 to 0.066)	2,615	16.793 (16.155 to 17.449)	27	0.173 (0.114 to 0.252)	10	0.064 (0.031 to 0.118)
**2015—total**	**9**	**0.006 (0.003 to 0.01)**	**3,271**	**2.009 (1.941 to 2.079)**	**77**	**0.047 (0.037 to 0.059)**	**29**	**0.018 (0.012 to 0.026)**
0–4 years old	2	0.012 (0.001 to 0.043)	261	1.552 (1.37 to 1.752)	28	0.167 (0.111 to 0.241)	1	0.006 (0 to 0.033)
5–14 years old	1	0.003 (0 to 0.017)	40	0.121 (0.086 to 0.164)	1	0.003 (0 to 0.017)	1	0.003 (0 to 0.017)
15–44 years old	1	0.001 (0 to 0.007)	185	0.248 (0.213 to 0.286)	3	0.004 (0.001 to 0.012)	7	0.009 (0.004 to 0.019)
45–59 years old	1	0.005 (0 to 0.025)	179	0.813 (0.698 to 0.941)	5	0.023 (0.007 to 0.053)	6	0.027 (0.01 to 0.059)
60 + years old	4	0.025 (0.007 to 0.064)	2,606	16.221 (15.605 to 16.857)	40	0.249 (0.178 to 0.339)	14	0.087 (0.048 to 0.146)

ICD-10, International Statistical Classification of Diseases and Related Health Problems 10th Revision; J00-J06, Acute upper respiratory infections; J09-J18, Influenza and pneumonia; J20-J22, Other acute lower respiratory infections; J85-J86, Suppurative and necrotic conditions of lower respiratory tract.

The yearly burden of disease ranged between 99864 (97672–102093) to 119300 (116897–121712) DALYs equivalent to 6.39 (6.36–6.43) to 7.56 (7.52–7.61) DALYs per 1000 population (Table 5). In every year studied 99% of DALYs were attributed to YLLs mainly due to premature mortality by influenza and pneumonia in population under 5 years-old and over 60 years-old (Table 6). There were no significant differences in the burden of disease estimations with Coale and Demeny model life table West and WHO 2011–2015 life tables estimates for Ecuador.

**Table 5:** Estimated burden of acute respiratory infections in Ecuador in the period 2011–2015 (ICD10 codes: J00—J22, J85—J86).

Year	population at risk—n	cases—n	deaths—n	YLD—n (95% CI)	YLL—n (95% CI)	DALY—n (95% CI)	DALY—rate (95% CI)*
2011	15266431	3655404	3219	1936 (1616–2262)	97928 (95770–100126)	99864 (97672–102093)	6.54 (6.5–6.58)
2012	15520973	3321199	3758	1712 (1436–2000)	114308 (111989–116663)	116020 (113688–118393)	7.48 (7.43–7.52)
2013	15774749	3062091	3871	1544 (1297–1802)	117756 (115356–120165)	119300 (116897–121712)	7.56 (7.52–7.61)
2014	16027466	2459681	3521	1122 (942–1310)	107123 (104810–109427)	108245 (105927–110555)	6.75 (6.71–6.79)
2015	16278844	2341111	3388	1015 (849–1187)	103072 (100819–105319)	104087 (101838–106349)	6.39 (6.36–6.43)

ICD-10, International Statistical Classification of Diseases and Related Health Problems 10th Revision; J00-J06, Acute upper respiratory infections; J09-J18, Influenza and pneumonia; J20-J22, Other acute lower respiratory infections; J85-J86, Suppurative and necrotic conditions of lower respiratory tract.

* Rates per thousand population.

**Table 6:** Total DALY per sex and age group attribuitable to acute respiratory infections in Ecuador in the period 2011–2015.

Age group	2011			2012			2013			2014			2015		
	Total	Male	Female	Total	Male	Female	Total	Male	Female	Total	Male	Female	Total	Male	Female
0–4 years old	14007	6910	7097	14277	7043	7234	11737	5790	5947	11337	5592	5745	9398	4636	4762
5–14 years old	2758	1361	1397	2562	1264	1298	2580	1273	1307	1871	923	948	1504	742	762
15–44 years old	8323	4105	4218	7252	3577	3675	8999	4439	4560	7297	3599	3698	6123	3020	3103
45–59 years old	6072	2995	3077	6488	3200	3288	8890	4385	4505	6800	3354	3446	5879	2900	2979
60+ years old	68697	33882	34815	85428	42134	43294	87097	42957	44140	80946	39923	41023	81199	40048	41151
Total	99857	49253	50604	116007	57218	58789	119303	58844	60459	108251	53391	54860	104103	51346	52757

The average annual losses due to indirect cost in current US dollars equals US$152.16 million (±19.6 millions), equivalent to 0.164% of the Gross Domestic Product of Ecuador. Total productivity losses in the five-year period studied accounts US$760.8 million. (Table 7)

**Table 7:** Estimated productivity losses (in current US dollars) due to acute respiratory infections in Ecuador 2001–2015.

Year	Ambulatory cases	Hospital admissions	Deaths	Total
2011	$ 93.071.796	$ 6.392.363	$ 73.032.856	$ 172.497.014
2012	$ 84.377.604	$ 6.782.648	$ 66.720.152	$ 157.880.403
2013	$ 77.548.236	$ 8.126.369	$ 85.583.928	$ 171.258.533
2014	$ 62.172.349	$ 7.101.019	$ 67.234.336	$ 136.507.704
2015	$ 59.205.873	$ 6.537.248	$ 56.913.464	$ 122.656.585
Total	$ 376.375.857	$ 34.939.646	$ 349.484.736	$ 760.800.239

## Discussion

Most of the available estimations of burden of pneumonia, acute respiratory diseases and other infectious diseases, have been done through methodologies based on analysis of systematic reviews, expert opinion, literature reviews and data extrapolation [19–21]. In 2013, Savy et al. performed a systematic review to estimate the burden of influenza in Latin America and the Caribbean. This group stated that pneumonia and influenza related deaths are most common in population age groups under5 years-old and over 60-years-old, reporting that influenza and pneumonia deaths in Ecuador were the highest in Latin America (14.4%) for the year 2003 in children under 5 years-old. Finally, they stated that underreporting and scarce information impede accurate estimation of impact of influenza in the region of Latin America and the Caribbean [22].

Available data about DALYs in Ecuador for upper and lower respiratory infections in 2015 obtained from the Institute for Health Metrics and Evaluation Global Health Data Exchange web page (IHME) [23] reports 184 053.03 DALYs (95% CI 213 970.98–162 755.42). The differences obtained between the results reported in the present study (104 103 DALY) and the estimations obtained by the IHME could be attributed to methodological differences. The overlapping clinical syndromes caused by the etiologic agents of acute respiratory infections make it difficult to assign a specific etiology based on the clinical presentations. Our approach using both hospitalized cases and ambulatory cases databases provide an estimation of burden without adjustment for other non-specific causes of morbidity and mortality. Thus, obtaining reliable population-based estimates for disease burden of respiratory infections remains a challenge.

The two highest drivers of the burden of acute respiratory infections are premature deaths in the populations over 60 years-old and under 5 years-old, which produces a high number of YLL. This “U-shaped” distribution of death rates by age group is concordant with the situation reported by Lara-Oliveros et al. [24]in a low income district in Bogota, Colombia, and also compares to reports from Brazil and India [25,26]. Worldwide, acute respiratory infections were responsible for about 1.9 million pediatric deaths in 2000 [27,28]. According the Forum of International Respiratory Diseases, more than 4 million deaths annually are produced by acute respiratory infections in developing countries. This forum attributes risk factors as living in crowded conditions, malnutrition, lack of immunization, HIV and exposure to tobacco or indoor smoke [1,2]. Public health decisions concerning acute respiratory diseases should be focused in these two age groups. While partial seasonal patterns in mortality and hospitalizations were found in this study, seasonal effect and molecular characterization of circulating viruses in ambulatory consultations should be investigated in further research. A recent study on the etiology of severe pneumonia in Ecuadorian children described the association of respiratory syncytial virus, metapneumovirus, and adenovirus with severe infections [29]. In temperate regions, public health decisions about influenza immunization are defined upon viral characterizations for each region. In tropical countries it is not clear if it is necessary to follow a seasonal pattern or not. New evidence about recommendations of vaccine use in tropical regions could be implemented in Ecuador [30–32]. Seasonal influenza vaccine, conjugate pneumococcal vaccine and Pentavalent Vaccine (DPT-HepB-Hib) were administered regularly in 2007 as part of the national vaccination program [33]. Currently, the Ecuadorian vaccination scheme includes programmed immunization with these agents [34]. According to official reports, vaccine coverage has declined since 2014 until 2017 in all age groups. For instance, reported coverage in children from 6 to 11 months of age fell from 79% in the 2014–2015 period, to 46% in the 2016–2017 period. For children of 1 to 3 years-old, coverage declined from 93 to 65% and in pregnant women, it declined from 72 to 43%. In people older than 65 years-old, it declined from 100% of coverage for 2014–15 to 82% in the 2015–16 period. There is no data for this specific age group for the 2016–17 period [8]. A limitation in this study was that data before 2007 is not available to compare the impact of these interventions in matters of disease burden.

Availability of public databases allows prompt use of data to assess epidemiological variables in population and decisions in public health. Existing data of acute upper respiratory infections are always underestimated because of nonspecific clinical presentation and a perceived lack of need of medical attention for a benign illness.

Acute respiratory infections are directly linked to prescribed or auto-prescribed consumption of drugs. The demand for generic and brand name drugs is highly driven by non-steroidal anti-inflammatory drugs (NSAIDs), especially by those recommended for the symptoms of cold and flu [2]. In a short unpublished analysis, during 2012 to 2014, Ecuador spent more than 29.5 million USD in flu and cold medication (NSAIDs + nasal decongestant + antihistamine) representing more than 6.4 million units sold during those years. The consumption of treatment drugs is determined essentially by 1) doctors’ prescription given in ambulatory attention, 2) hospitalization and rehabilitation, and/or by 3) the user (auto-medication—auto-prescription [35]. Access to these drugs is also regulated by the economic status of the population, with the poorer group spending the most in out of pocket expenses [36]. In further research, direct and indirect costs related with acute respiratory infections should be estimated.

Direct costs could not be estimated in this study because the health system in Ecuador does not have a billing system or register of direct expenses generated by medical consultation. However, our estimation of indirect costs could be an initial approach to the economic impact that acute respiratory infections produces in the economy of Ecuador.

## Conclusions

Periodic analyses of the burden of diseases can stimulate the implementation of evidence-based prevention and control policies to tackle acute respiratory infections in Ecuador specially influenza and pneumonia cases at extreme ages of life. Implementation of methodic processes of estimation should be encouraged in multiple scenarios.

National immunization programs are the first intervention that must be maintained in terms of coverage and quality. Activity surveillance and virological characterization of respiratory viruses especially at primary care settings need to be implemented to complement information of acute respiratory infections. Finally, widespread awareness in health services in the country about impact, management and report of acute respiratory infectious should be permanent.
